# High expression of GEM and EDNRA is associated with metastasis and poor outcome in patients with advanced bladder cancer

**DOI:** 10.1186/1471-2407-14-638

**Published:** 2014-08-30

**Authors:** Jens Reumert Laurberg, Jørgen Bjerggaard Jensen, Troels Schepeler, Michael Borre, Torben F Ørntoft, Lars Dyrskjøt

**Affiliations:** Department of Molecular Medicine, Aarhus University Hospital, Brendstrupgaardsvej 100, 8200 Aarhus N, Denmark; Department of Urology, Aarhus University Hospital, Brendstrupgaardsvej 100, 8200 Aarhus N, Denmark

**Keywords:** Bladder cancer, Metastasis, Outcome, GEM, EDNRA

## Abstract

**Background:**

The standard treatment for non-metastatic muscle-invasive bladder cancer (stages T2–T4a) is radical cystectomy with lymphadenectomy. However, patients undergoing cystectomy show metastatic spread in 25% of cases and these patients will have limited benefit from surgery. Identification of patients with high risk of lymph node metastasis will help select patients that may benefit from neoadjuvant and/or adjuvant chemotherapy.

**Methods:**

RNA was procured by laser micro dissection of primary bladder tumors and corresponding lymph node metastases for Affymetrix U133 Plus 2.0 Gene Chip expression profiling. A publically available dataset was used for identification of the best candidate markers, and these were validated using immunohistochemistry in an independent patient cohort of 368 patients.

**Results:**

Gene Set Enrichment Analysis showed significant enrichment for e.g. metastatic signatures in the metastasizing tumors, and a set of 12 genes significantly associated with lymph node metastasis was identified. Tumors did not cluster according to their metastatic ability when analyzing gene expression profiles using hierarchical cluster analysis. However, half (6/12) of the primary tumor clustered together with matching lymph node metastases, indicating a large degree of intra-patient similarity in these patients. Immunohistochemical analysis of 368 tumors from cystectomized patients showed high expression of GEM (P = 0.033; HR = 1.46) and EDNRA (P = 0.046; HR = 1.60) was significantly associated with decreased cancer-specific survival.

**Conclusions:**

GEM and EDNRA were identified as promising prognostic markers for patients with advanced bladder cancer. The clinical relevance of GEM and EDNRA should be evaluated in independent prospective studies.

## Background

Bladder cancer is the 4th most common cancer in men and the 11th most common cancer in women [1]. Patients with non-muscle-invasive bladder cancer (NMIBC) are predominantly treated with transurethral resection of the bladder in combination with Bacillus Calmette-Guerin (BCG) or Mitomycin C. Cystectomy is offered if local control cannot be maintained. Recently, treatment of NMIBC has shifted towards a more aggressive approach based on EORTC risk scores, resulting in more patients receiving cystectomy [2, 3]. The standard treatment for non-metastatic muscle-invasive bladder cancer (MIBC) (stages T2–T4a) is radical cystectomy with lymphadenectomy [4]. Patients with immobile tumors (T4b) receive chemotherapy– sometimes followed by salvage cystectomy or radiotherapy [5]. Five-year cancer-specific survival for patients with MIBC is 65% following cystectomy and neoadjuvant chemotherapy increases the 5-year survival with 6–8% but is, for now, not standard treatment in all clinical settings [6, 7].

Patients undergoing cystectomy show metastatic spread in 25% of cases [8], and these patients will have limited benefit of surgery. Identification of patients with high risk of lymph node metastasis could help identify patients that would benefit from neoadjuvant chemotherapy. Therefore, identification of metastatic disease (to lymph nodes or distant organs) prior to cystectomy is of high importance. Previously, several studies have focused on studying molecular markers to identify metastatic risk or ability based on analysis of the patient’s primary tumor. Key players in the DNA-damage-response and cell-cycle machinery (e.g. p53, Rb, p21, p16, Tip60) have been investigated by immunohistochemistry, but none of the markers have shown significant power in validation studies to reach the clinic [9–12]. More recently, gene-expression signatures have revealed promising results but have not yet been validated in prospective patient cohorts [13, 14]. Smith *et al.* reported a 20 gene signature in the primary tumor for predicting lymph node metastasis based on three different cohorts, making it the first study in MIBC where the gene signature was validated in an independent patient cohort [15]. Patients with high relative risk (1.74) and low relative risk (0.70) of node positive disease could be identified. In other disease like e.g. breast cancer, metastatic capacity of the primary tumors has been studied intensely, and several gene expression signatures for predicting metastatic outcome have been develop and successfully validated [16–19].

Here we laser micro dissected primary bladder tumors and corresponding lymph node metastases and performed microarray gene expression profiling of the procured cells. We compared gene expression patterns in primary bladder tumors with and without metastatic disease and by including previously published data from Riester *et al.* [20] we identified a panel of 12 transcripts significantly associated with disease outcome. The prognostic value of GEM (GTP binding protein overexpressed in skeletal muscle) and EDNRA (endothelin receptor type A) were successfully validated in an independent patient cohort using tissue microarrays (TMAs).

## Methods

### Patients and follow-up

Written informed consent was obtained from all patients and the study was approved by the Central Denmark Region Committees on Biomedical Research Ethics (1994/2920). All patients were cystectomized at Department of Urology at Aarhus University Hospital between 1998 and 2008, and surviving patients had at least 36 months of follow-up, and were censored after a maximum of 96 months. Tumor stage was determined using the American Joint Committee on Cancer recommendations from 2002 and WHO 2004 classification was used to determine tumor grade. All patients were clinically free of metastasis before surgery and no patients received neoadjuvant or adjuvant treatment in terms of chemotherapy or radiotherapy.

### Laser micro dissection, RNA extraction and microarray analysis

All patient specimens collected at the time of surgery were split into tissue for pathology and tissue for the biobank. Tissue for the biobank was embedded in Tissue-Tek® O.C.T™ Compound and snap frozen in liquid nitrogen before storage at -80°C. Sections were examined by a genitourinary pathologist to identify carcinoma cell content. Following, cresyl violet stained tissue was microdissected using the PALM laser microbeam system. RNA extraction was performed using RNeasy Micro Kits (Qiagen) according to manufacturer protocols. RNA quality was assessed using an Agilent Bioanalyzer 2100 (RIN: 2.4-8.8; median 5.9). Total RNA was amplified and converted to cDNA using Nugen Pico-RNA system. The two-round amplification kit is optimized to amplify low volumes and poor quality RNA for Affymetrix array analysis. After amplification, the cDNA was fragmented and labeled using NuGen FL-Ovation kit, loaded onto the Affymetrix U133 Plus 2.0 Gene Chip according to the manufacturer’s protocol, and scanned using the Affymetrix 3000 7G Scanner.

### Microarray data analysis

Raw microarray data was normalized and intensity measures generated by RMA [21] using GeneSpring version 11 software. Unsupervised hierarchical cluster analysis of all transcripts with a variance above 1.5 was performed using Cluster 3.0 and Java tree-view software [22]. Gene Set Enrichment Analysis (GSEA) v2.07 software was used to test if previously published gene signatures and curated pathways were enriched in the data. We used the inbuilt KEGG, BIOCARTA, REACTOME, gene ontology, and oncogenic signatures in MsigDB database and supplemented with curated signatures containing “cancer”, “metastasis”, “cell cycle”, “repair”, “DNA damage”, and “hypoxia”. We used the default significance levels to test if significant enrichment was reached with normalized p-values below 0.05 and with false discovery rates below 0.25. A previously published dataset (GEO ID: GSE31684; U133 Plus 2.0 GeneChip) from laser microdissected tumors from 93 cystectomized patients was retrieved. A total of 69 patients were included in the analysis, after exclusion of all patients without reported lymph node status, and all node negative patients without 24 months of follow-up.

### Tissue microarray (TMA) analysis

Biopsies from a total of 368 tumors from cystectomy specimens and from 41 lymph node metastases were incorporated into a TMA. All tumors were reevaluated regarding T-stage and grade by the same uro-pathologist prior to placement on the TMA. The patients included and the TMA construction is described earlier [11].

### Immunohistochemistry and Western blotting

The immunohistochemichal staining procedure was carried out based on the EnVision + TM System HRP (Dako) as previously described [23]. Antibodies against GEM (Novus Biologicals # NBP1-58906) diluted 1:150 and against EDNRA (Abcam #ab76259) diluted 1:800 were used. The specificity of the antibodies against GEM and EDNRA was validated by Western blotting using T24 cell line essentially as described earlier [24].

### Scoring of IHC staining

A Hamamatsu Nanozoomer scanner (Hamamatsu Corporation, Hamamatsu City, Japan) was used to scan the TMA slides, and VIS visualization software (Visiopharm A/S, Hørsholm, Denmark) was used for visualization of IHC staining during scoring of the protein expression intensities. Percentage of positive carcinoma cells was scored on a continuous scale for each core, and optimal cut-off values were afterwards defined by ROC curves. Scoring was performed by two observers blinded to outcome. The first observer scored on a continuous scale, and the second scored according to the dichotomized cutoff value generated. Differences in the dichotomized scorings were reviewed and consensus was reached.

### Statistics

Comparisons between the metastatic and non-metastatic groups were performed using two-sided t-test statistics. Categorical data was compared in univariate analysis using the χ2 test and censored data was compared using log-rank test. Hazard ratios (HR) were estimated using Cox proportional hazard models. Multivariate analysis was performed separately for each biomarker including only significant clinical parameter from the univariate analysis. All analyses were performed using STATA (version 11).

## Results

For gene expression profiling we selected 18 primary tumors and 12 matched lymph node metastases from 18 patients with bladder cancer. Ten patients had at least one lymph node metastasis at time of cystectomy, and 6 patients died of bladder cancer. Clinical and histopathological information for each patient is listed in Table 1.

**Table 1:** **Clinical and histopathological information for each patient used for gene expression profiling**

Patient	Gender	T-stage	N status	Relapse	Dead of Bladder cancer	Time to relapse (months)	Follow up (months)
2211	Man	4a	Positive	No	No		24
1599	Woman	1	Positive	No	No		22
2114	Man	3b	Positive	Yes	Yes	9	61
2117	Man	3b	Positive	No	No		11
2130	Man	1	Positive	Yes	Yes	14	16
2163	Man	2	Positive	No	No		65
2180	Man	3	Positive	Yes	Yes	18	30
2207	Man	4a	Positive	Yes	Yes	9	22
2249	Woman	1	Positive	Yes	Yes	3	13
2237	Woman	2	Positive	No	No		31
1956	Man	1	Negative	No	No		66
1930	Man	1	Negative	No	No		61
1940	Woman	2	Negative	No	No		61
1743	Man	1	Negative	Yes	Yes	40	57
2036	Man	2	Negative	No	No		77
1874	Man	3b	Negative	No	No		63
1607	Woman	2	Negative	No	No		60
1956	Man	1	Negative	No	No		61

### Molecular subgroup analysis

**Figure 1:**
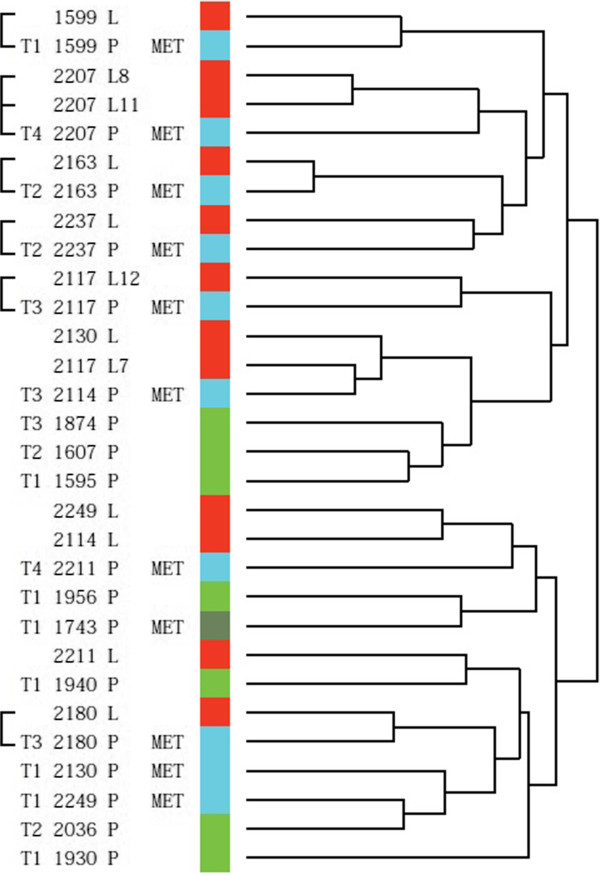
**Unsupervised hierarchical cluster analysis of all samples.** Square brackets are used when the coupled tumor and metastasis cluster together. Green color represents a primary non-metastasizing tumor. Dark green represents a primary non-metastasizing tumor which later develops lymph node metastases in the abdomen. Blue color represents a primary metastasizing tumor. Red color represents a lymph node metastasis.

Initially, data was filtered, selecting only transcripts with a variance above 1.5 across all samples (11046 transcripts). We performed unsupervised hierarchical cluster analysis to investigate if tumors clustered based on stage or metastatic abilities, and if lymph nodes showed a high degree of similarity to the matched primary tumors (Figure 1). Cluster analysis separated the tumors into two main clusters; one cluster (cluster A) contained seven primary metastasizing tumors, three primary non-metastasizing tumors, and eight lymph nodes, and among these were six of the seven matched pairs. The other cluster (cluster B) contained five primary non-metastasizing tumors, five metastasizing primary tumors, and four lymph nodes. Seven of the lymph nodes clustered together with their matched primary tumor, indicating a large degree of intra-patient similarity in these patients. However, the overall expression patterns did not show significant separation of the tumors based on metastatic ability. Most of the muscle-invasive tumors clustered together in cluster A – as expected.

### Gene set enrichment analysis (GSEA)

To investigate the differences between the metastatic and non-metastatic tumors more specifically, we applied GSEA for investigating enrichment for previously published signatures regarding key elements in the metastatic process together with enrichment for pathway elements (Table 2). Interestingly, all signatures regarding extracellular function, metastasis, hypoxia, proliferation, and survival were exclusively enriched in metastatic tumors while all signatures regarding repair and cell cycle were enriched in non-metastatic tumors. Cell signaling was primarily enriched in metastatic tumors while metabolism was primarily enriched in non-metastatic tumors. In addition, we investigated enrichment for previously published signatures comparing primary tumors and metastasis [25–27]; both signatures containing tumors from many different tissues were significantly enriched in our dataset (Ramaswamy *et al.*, P = 0.02 and Daves *et al.*, P = 0.03), while the signature from metastatic malignant melanoma was borderline significantly enriched (Daves *et al.*, P = 0.06).

**Table 2:** **GSEA of published signatures in MsigDB**

	Enriched in metastatic tumors	Enriched in non-metastatic tumors
Extracellular function	10	0
Metastasis	7	0
Proliferation and survival	7	0
Hypoxia up	1	0
Cell signaling	20	6
Metabolism	8	13
Hypoxia down	0	1
Repair	0	15
Cell cycle	0	33
Others	17	32

### Matched-pair analysis

**Figure 2:**
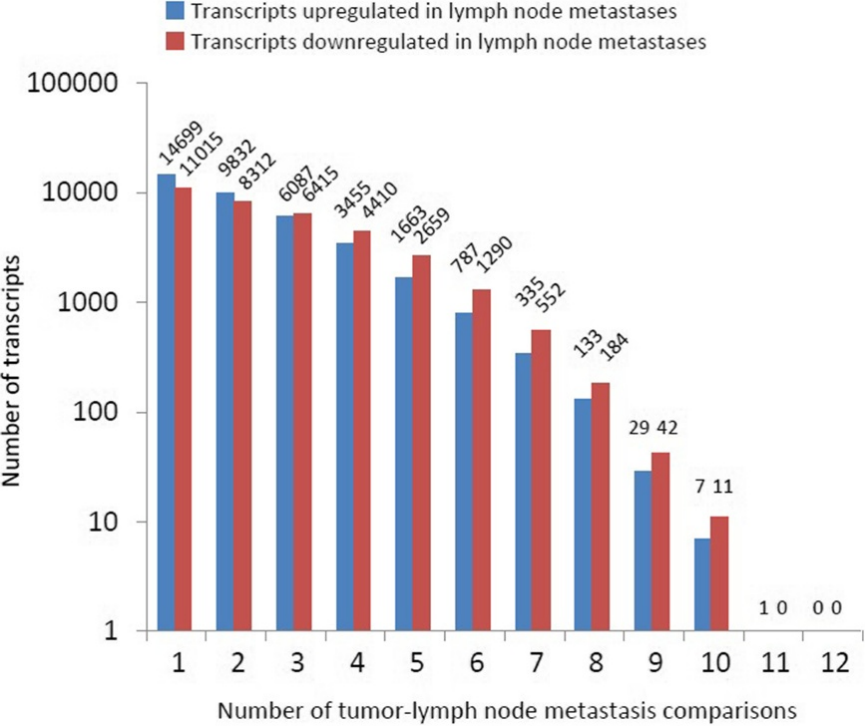
**Tumor heterogeneity measures.** The distribution of transcripts with more than two-fold difference in tumor-metastasis pair comparisons. Two lymph node metastases were included from two patients resulting in 12 comparisons in total.

We used the paired tumors and lymph node metastases to investigate the intra- and inter-patient similarity. When comparing differences in transcript levels between the matched primary tumors and metastases using two-fold difference as cut-off, we did not find any transcripts that were differentially expressed in all 12 tumor-lymph node comparisons (Figure 2). *MMP2* was the only gene that was down-regulated in 11 lymph node metastases, while 18 transcripts were up or down regulated in 10 lymph node metastases. In general, as observed in the cluster analysis, the patients show a large heterogeneity in expression patterns between primary tumors and lymph node metastases. Using Ingenuity Pathway Analysis we did not identify any general pathway changes between primary tumors and lymph node metastases, probably because of this large heterogeneity observed between patients.

### Identification of markers associated with outcome

**Figure 3:**
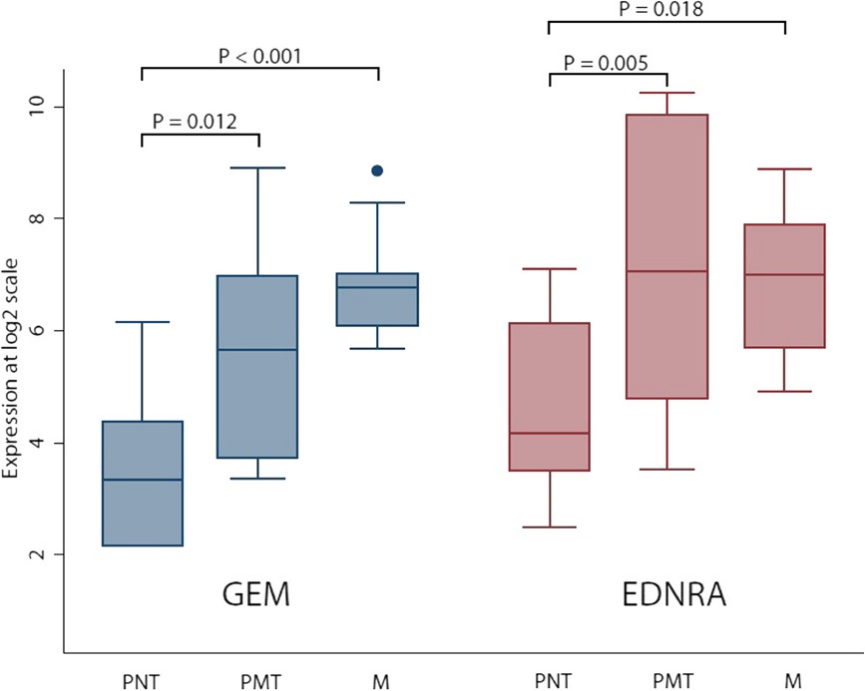
**Differences in** ***GEM*** **and** ***EDNRA*** **expression in primary non-metastasizing tumors (PNT), primary metastasizing tumors (PMT), and lymph nodes metastases (M).**

**Figure 4:**
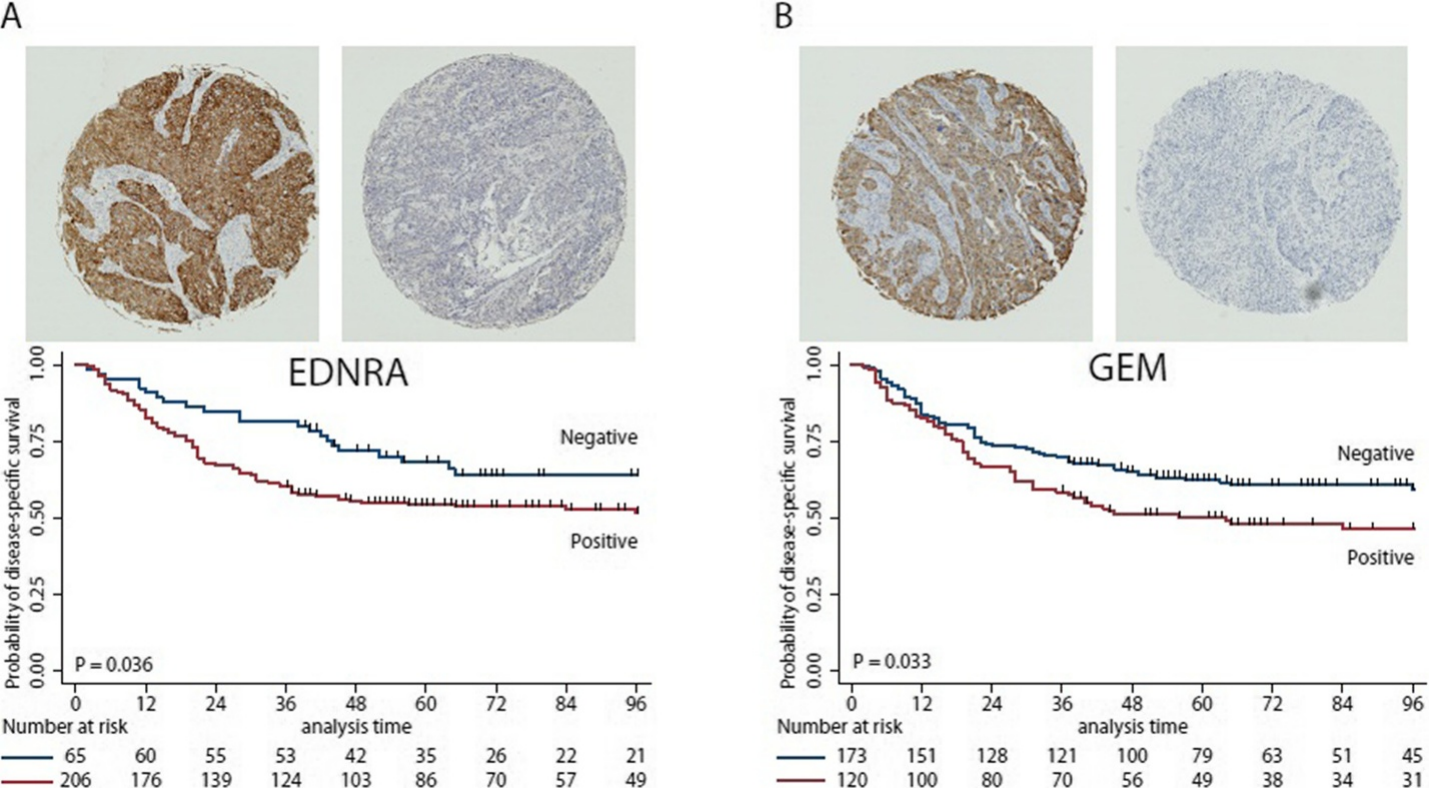
**EDNRA (A) and GEM protein (B) expression in the TMA validation cohort.** Top: Staining pattern of a positive and a negative core of EDNRA and GEM. Bottom: Kaplan-Meier survival curves of disease specific survival as a function of marker expression in the patient cohort.

Because of the large heterogeneity observed and because of the limited sample size we included a previously published dataset for delineation of markers associated with outcome (GEO ID: GSE31684). The dataset contained Affymetrix U133 Plus 2.0 GeneChip data from 69 patients with known lymph node status and at least 24 months of follow-up if no lymph node metastasis was present at surgery. Separately, for both datasets, we delineated transcripts associated with the presence or absence of metastasis; only transcripts with a mean fold change difference > 2 and with a P < 0.05 (student’s t-test) were selected. Twelve transcripts up-regulated in metastasizing tumors passed our selection criteria in both datasets (Table 3). We selected *EDNRA* and *GEM* (Figure 3) for further validation using immunohistochemistry (IHC). For this we used a tissue microarray containing 409 core biopsies from both primary tumors (n = 368) and lymph node metastases (n = 41). Both GEM and EDNRA protein expression was localized in the cytoplasm of the cells, and no staining was observed in normal urothelium or connective tissue cells. IHC scoring was performed by two observers independently, with an inter-observer agreement of 0.70 (GEM) and of 0.81 (EDNRA), using Cohen’s kappa. The clinical and histopathological characteristics for the patients included in this cohort are listed in Table 4. High expression of GEM (P = 0.033; HR = 1.46) and EDNRA (P = 0.046; HR = 1.60) were significantly associated with decreased cancer-specific survival (Figure 4). Furthermore, after performing multivariate analysis high EDRNA expression showed significantly association with decreased cancer-specific survival (P = 0.046), while GEM showed no significance (P = 0.11). Finally we investigated the similarity in protein expression between matched primary tumors and lymph node metastases; 94% of the lymph nodes showed similar expression as in the primary tumors for EDNRA and 71% for GEM.

**Table 3:** **Transcripts significantly up-regulated in metastasizing tumors in both cohorts**

	Non-metastatic vs metastatic tumors		Lymph node metastasis vs non-metastatic tumors		Non-metastatic vs metastatic tumors (Riester et al.)	
Transcript	p-value	FC	p-value	FC	p-value	FC
*COL6A2*	0.0397	1.0967	0.7515	0.1176	0.0461	1.7263
*LMCD1*	0.0248	1.1631	**0.0036**	1.7576	0.0196	1.7212
*FZD1*	0.0287	1.5193	0.0878	1.1318	0.0055	1.0648
*MITF*	0.0364	1.6083	0.4593	-0.3003	0.0164	1.0783
*EDNRA*	0.0051	1.6613	**0.0181**	1.0262	0.0177	1.4840
*EBF1*	0.0211	1.7592	**0.0168**	1.8477	0.0149	1.0386
*TPST1*	0.0199	1.7953	0.1975	0.8709	0.0318	1.1064
*AEBP1*	0.0242	2.2697	**0.0077**	1.6563	0.0072	3.0447
*PALLD*	0.0344	2.3131	0.1163	0.9763	0.0104	1.4558
*GEM*	0.0121	2.3136	**0.0000**	3.2533	0.0219	1.5247
*PXDN*	0.0044	3.1464	**0.0042**	1.8611	0.0356	1.9232
*KITLG*	0.0110	3.3621	0.0537	1.6857	0.0323	1.1616

FC = Log 2 fold change differences.

Bold indicates significant p-values when comparing lymph node metastasis and non-metastatic tumors.

**Table 4:** **Univariate and multivariate Cox regression analysis of disease specific survival as function of molecular markers**

		Univariate analysis	Multivariate analysis including EDNRA	Multivariate analysis including GEM
Nr. of patients	368			
Median Follow-up months (range)	62 (2–96)			
Age median (range)	64 (39–79)	HR = 1.01 (P=0.47)		
Sex		**HR = 1.56 (P=0.007)**	**HR = 1.62 (P=0.015)**	**HR = 1.45 (P=0.048)**
Men	268			
Women	100			
T-stage		**HR = 1.59 (P<0.001)**	**HR = 1.28 (P=0.049)**	HR = 1.22 (P=0.070)
T1	43 (12%)			
T2	129 (35%)			
T3	146 (40%)			
T4	50 (13%)			
Lymph node metastases		**HR = 3.98 (P<0.001)**	**HR = 3.82 (P<0.001)**	**HR = 3.55 (P<0.001)**
N0	278 (76%)			
N1-3	89 (24%)			
Grade		HR = 1.18 (P=0.78)		
Low grade	8 (2%)			
High grade	360 (98%)			
EDNRA		**HR = 1.60 (P=0.046)**	**HR = 1.63 (P=0.042)**	
High	206 (76%)			
Low	65 (24%)			
GEM		**HR = 1.46 (P=0.032)**		HR = 1.33 (P=0.11)
High	173 (59%)			
Low	120 (41%)			

Values in bold indicate significant uni- and multivariate analysis (P<0.05).

## Discussion

The risk of recurrence and later metastasis following cystectomy is as high as 50% [28] and most patients will ultimately succumb to the disease following recurrence [29]. Therefore, early detection of metastasis and prediction of recurrence risk following cystectomy could ultimately improve survival as better treatment regimens could be applied. The aim of this study was to identify markers of lymph node metastasis at (before) cystectomy. We compared gene-expression profiles from 10 primary bladder tumors with 12 matched lymph node metastases and eight primary tumors without metastasis to identify markers associated with metastatic disease, and to test similarity between lymph node metastases and matched primary tumors. Overall, we found no large difference in gene expression between the two patient groups. Furthermore, we found that primary tumors and corresponding lymph node metastases showed comparable gene expression profiles in half of the cases. The reason for this lack of overall difference between the groups may be caused by tumor heterogeneity, minor sub clones responsible for metastatic ability, and also by inclusion of tumors of different stages (T1-T4). Gene set enrichment analysis (GSEA) was used to investigate biological differences between metastasizing and non-metastasizing tumors. Interestingly, signatures associated with “metastasis”, “extracellular function”, “proliferation and survival”, and “cell signaling” were significantly enriched in the metastasizing tumors while signatures associated with “metabolism”, “cell cycle” and “DNA repair” were associated with non-metastatic tumors – indicating that the overall biological process may be different in the two tumor groups. However, due to the large heterogeneity we were not able to identify general molecular differences between lymph node metastases and primary tumors.

The tumor heterogeneity (intra and inter) may make marker identification difficult, and consequently we included additional patient samples from a previously published dataset [20] for delineating significant markers of outcome. The panel of 12 genes that were significant in both datasets contained *GEM* and *EDNRA*. These genes were selected for further validation based on significance, difference in expression, expression level, and based on antibody availability. We found no overlap between our 12 genes and the 21-gene metastasis signature reported by Smith et al. previously [15], which may reflects multiple factors like cohort heterogeneity and size, and differences in sampling (laser micro dissection vs bulk tumor analysis). We found high expression of GEM and EDNRA to be significantly associated with a decrease in cancer-specific survival, when analyzing the protein expression on a cohort of 368 patients. Furthermore, high EDNRA was significantly associated with decreased cancer-specific survival in multivariate analysis.

The possible functional roles of EDNRA (endothelin receptor type A) and GEM (GTP binding protein overexpressed in skeletal muscle) in cancer progression and metastasis are currently unclear. EDNRA and GEM have not been associated with disease outcome and cancer outcome. GEM is a small GTP-binding protein that plays a role in regulating Ca^2+^ channel expression at the cell surface [30]. Furthermore, it is involved in cytoskeletal remodeling in interphase cells and is a spindle-associated protein required for prober mitotic progression [31]. EDNRA is a G-protein coupled receptor for endothelins and it is expressed on vascular smooth-muscle cells and on heart, kidney, and neuronal cells [32].

This study included a limited number of tumors in the initial characterization of tumor subgroups, and although we isolated carcinoma cells in primary tumors and lymph node metastases using laser-micro dissection, the patient cohort may still be too small to draw firm conclusion regarding molecular subgroups and differences between primary tumors and metastatic lesions. The strength of our approach is the inclusion of matched lymph node metastasis in the selection of candidate markers for metastasis, and this is to our knowledge the first study of bladder cancer that compare the lymph nodes to the primary tumors.

Recently, large intra-tumor heterogeneity of several cancer types has been reported [33–35]. A recent study of clear cell renal cell carcinomas showed significant molecular heterogeneity using whole-exome sequencing of multiple tumor areas [36]. As small cellular sub-clones may be responsible for the disease progression and metastasis it may be difficult to identify any good molecular markers of outcome by analyzing the bulk tumors. Other studies of tumor metastasis in mice have shown limited overlap in genomic alterations (about 9%) between primary tumors and metastases [37], indicating that metastatic lesions probably propagate from small sub-populations in the primary tumors. Intra-tumor heterogeneity has so far not been addressed in detail in bladder cancer. However, Li *et al.* [38] performed whole-exome sequencing of 66 individual cells from a single muscle invasive tumor, and identified large variation in mutant genes between the cells. Other groups [39, 40] have recently shown that muscle invasive bladder cancers belong to 4–5 distinct molecular subgroups. Consequently, future studies of prognostic markers for patients with advanced bladder cancer should include large patient cohorts, stratification according to overall tumor subgroup and sub-clonal analysis to compensate for the large inter and intra tumor heterogeneity for these patients.

## Conclusion

We observed a high degree of heterogeneity between primary tumors with and without metastases, and between paired samples of primary tumors and associated lymph-node metastases. GEM and EDNRA were identified to be promising prognostic markers for patients with advanced bladder cancer. The clinical relevance of GEM and EDNRA should be evaluated in independent prospective studies.
